# Solid-stemmed spring wheat cultivars give better androgenic response than hollow-stemmed cultivars in anther culture

**DOI:** 10.1007/s11627-016-9793-2

**Published:** 2016-12-01

**Authors:** Dorota Weigt, Angelika Kiel, Jerzy Nawracała, Mateusz Pluta, Agnieszka Łacka

**Affiliations:** 1 Department of Genetics and Plant Breeding, Poznan University of Life Sciences, Poznan, Poland; 2 Department of Mathematical and Statistical Methods, Poznan University of Life Sciences, Poznan, Poland

**Keywords:** Androgenesis, Haploid, Spring wheat, Solid stem

## Abstract

Solid-stemmed spring wheat cultivars (*Triticum aestivum* L.) are resistant to the stem sawfly (*Cephus cinctus* Nort.) and lodging. Anthers of 24 spring wheat cultivars with varying content of pith in the stem were used in the experiment. All were classified into three groups: solid, medium–solid and hollow stems. There was considerable influence of the cultivar on callus formation and green plant regeneration. The highest efficiency of green plant regeneration (24%) was observed for the solid-stemmed AC Abbey cultivar. There was no regeneration from the explants of four cultivars: CLTR 7027, Alentejano, Marquis and Bombona. Principal component analysis showed no differences between the cases under observation (callus induction and green plant regeneration) in their response to pre-treatment temperatures (4 and 8°C). The examination of the effects of various auxin types in the induction medium on callus formation and green plant regeneration revealed that the strongest stimulation of these processes was observed in the C17 medium with 2,4-D and dicamba. The efficiency of callus formation and green plant regeneration was greater in solid-stemmed cultivars than in hollow-stemmed cultivars.

## Introduction

Solid-stemmed wheat is highly resistant to the wheat stem sawfly (*Cephus cinctus* Nort.) (Weiss and Morrill 1992; Hayat *et al.* 1995) and lodging (Clarke *et al.* 2002; Kong *et al.* 2013). Furthermore, stem solidness improves yield under water-limited conditions by large storage capacity for water-soluble carbohydrates (Pierre *et al.* 2010). It would be beneficial to introduce genes determining the solid stem trait in well-yielding Polish varieties. The use of androgenesis in the breeding program would shorten the time needed for the development of new lines with a solid stem.

*In vitro* culture techniques give a possibility to obtain new genetic variability and intensify the process of breeding new varieties of wheat. By increasing the efficiency of haploid plant regeneration and obtaining doubled haploid lines (DH) from them, the breeding cycle would be significantly reduced, particularly in the case of self-pollinated crops such as wheat. It is much easier to analyse the traits of homozygous DH lines, since the phenotype of these plants corresponds to their genotype (Baenziger *et al.* 2001). Haploid wheat plants may be obtained by androgenesis in anther cultures, isolated microspore cultures, or by crossing with maize (wheat x maize system) and then by embryo culture (Verma *et al.* 1999; Niroula and Bimb 2009). The first of these methods has the greatest potential as, in theory, it allows the production of many haploid plants from one anther. However, androgenesis is limited by a strong impact of the donor plant genotype and the influence of external factors on the efficiency of regeneration of haploid green plants (Datta 2005; Forster *et al.* 2007). Therefore, in wheat breeding, some authors suggest the use of hybrids in which at least one parent has high androgenesis capacity (Bullock *et al.* 1982; Dağüstü 2008).

The aim of the present study was to analyse the relationship between the content of pith in the stem of spring wheat cultivars and their capacity to regenerate in anther cultures.

## Materials and Methods

### **Plant material**

The pith content in the stem was assessed at the Agricultural Experimental Farm in Dłoń, Poland, in 2012. Twenty-four spring wheat cultivars with varying content of pith in the stem were examined: four Polish cultivars with hollow stems (Arabella, Bombona, Ostka Smolicka and Parabola); 18 accessions from the National Small Grains Collection of the Agricultural Research Service of the United States Department of Agriculture, Aberdeen ID, USA; one cultivar from the Leibniz-Institut für Pflanzengenetik und Kulturpflanzenforschung—Gatersleben (Carola); and one cultivar (AC Abbey) from Dr. Ron DePauw (Agriculture and Agri-Food Canada (AAFC), Semiarid Prairie Agriculture Research Centre, Swift Current, Canada). All cultivars were grown under field conditions. The experiment design was a randomized block with three replicates. To estimate the degree of solidness, 50 individual plants were collected from each plot and cut in the middle of five consecutive internodes. The following 5-grade scale was used for evaluation: grade 1—hollow stem—0% filled by pith; grade 2, 25% filled by pith; grade 3, 50% filled by pith; grade 4, 75% filled by pith; and grade 5, 100% filled by pith. The total value of the content of pith in the stem was the sum of the values from all the internode cuts. For example, a plant with a hollow stem scored 5 points, while one with a completely filled stem scored 25 points.

To compare androgenic responses of cultivars with solid, medium–solid (medium) and hollow stems, all the cultivars were classified into three groups: 5–15, 15.1–20 and 20.1–25 points, respectively.

### **Preparation of donor plants and pre-treatment of donor spikes**

Donor plants for *in vitro* cultures were grown under field conditions in 2013 at the Experimental Nursery of the Department of Genetics and Plant Breeding, Poznań University of Life Sciences. For *in vitro* cultures, immature spikes were collected when the flag leaf emerged and when most microspores in the central region of the spikes were at a mid- or late-uninucleate stage. To verify the developmental stage of microspores, one or two anthers from each spike were examined microscopically using acetocarmine staining (Barnabas *et al.* 2001). Then, half of the spikes were placed in beakers with distilled water at 4°C and the other half at 8°C in the dark for 8 d in order to estimate the influence of different cold pre-treatment temperatures on the induction of androgenesis and plant regeneration.

### ***In vitro*culture**

After the cold pre-treatment, spikes were superficially sterilized with 4.85% (*w/v*) calcium hypochlorite (Sigma-Aldrich®, Poznan, Poland) for 4 min, then washed three times for 5 min with sterilized, distilled water. Anthers were excised from the spikes in a laminar airflow cabinet, and plated on 50 × 10 mm Petri dishes (NOEX, Komorniki, Poland) containing 8 mL of the solid induction medium. For callus induction, a C-17 medium (Wang and Chen 1983) with modification (Weigt *et al.* 2012) was used. The medium was solidified with 0.3% (*w/v*) Gelrite® (Sigma-Aldrich®, St. Louis, MO) and sterilized by autoclaving (121°C, 27 min and 121.6 kPa pressure). Two combinations of different growth regulators (added pre-autoclaving) were used: (1) 1.0 mg L^−1^ 2,4-dichlorophenoxyacetic acid (2,4-D ) and 1.0 mg L^−1^ 3,6-dichloro-2-methoxybenzoic acid (dicamba); or (2) 1.5 mg L^−1^ 2,4-D and 0.5 mg L^−1^ kinetin (Sigma-Aldrich®). Fifty anthers from a single spike were cultured in each Petri dish. Four combinations including pre-treatment and growth regulators were used. In each combination, 450 anthers of each cultivar were cultured. Petri dishes were sealed with Parafilm M® (Sigma-Aldrich®) and incubated at 28°C in the dark for 8 to 10 wk. Then the calluses were counted and transferred into 50 × 10 mm Petri dishes containing 8 mL of MS regeneration medium (Murashige and Skoog 1962) modified according to Weigt *et al.* (2012). The MS medium was solidified with 0.6% (*w/v*) agar (Sigma-Aldrich®) and autoclaved as mentioned above. After 4 to 6 wk young, green plantlets obtained on the regeneration medium were transferred to 150 mL conical flasks (Archem, Kamieniec Wroclawski, Poland) containing 30 mL of MS regeneration medium (described above) for further growth. Green plantlets with well-formed roots were transplanted to small pots with sterilized soil (2:1 peat/sand mixture), placed in a growth chamber and maintained at 24°C with a 16 h photoperiod at light intensity of about 250 μm m^−2^ s^−1^ provided by cool white fluorescent bulb (Zext, Sulejowek, Poland) for acclimatization.

### **Data analysis**

The experiments were analysed as a completely randomized designs. There were three replicates of each treatment. To estimate the effect of the cultivar and the influence of different pre-treatment temperatures and hormones in the induction medium, the following traits were determined: the efficiency of callus formation (CF), expressed as the number of calluses produced per 100 anthers; the efficiency of green plant regeneration (GPR), expressed as the number of green plants regenerated per 100 anthers; and the efficiency of albino plants regeneration (APR), expressed as the number of albino plants regenerated per 100 anthers.

Principal component analysis (PCA) was used to illustrate the relationship between the observed variables (callus formation, green and albino plant regeneration) and the replicates of the experiment representing different experimental combinations (Abdi and Williams 2010). This method is commonly used in biological studies to reduce the number of variables and to facilitate analysis by eliminating the autocorrelation between them. The analysis enables creation of a mathematical model in a virtual multidimensional space, and along the greatest diversity of the material analysed, it assigns a new set of values of linearly uncorrelated variables (principal components) and thus assigns new coordinates to individual observations. New variables were considered to be synthetic indicators, showing natural features in a spatial dimension. The sums of nine replications obtained for each case were analysed with a program package ggbiplot in R (R Development Core Team 2013).

## Results

### **Stem solidness**

The whole range of variability was observed among the plants tested, from solid-stemmed cultivars to those with a hollow stem. Of all the cultivars tested, eight were classified as solid-stemmed. The Polish cultivars used in the experiment were classified as hollow-stemmed. Some cultivars (e.g., Solid Straw Tuscan Varia, Americano 44D, Ruzynska II) that were characterized as solid-stemmed did not exhibit this trait under the environmental conditions of this study. Their scores of solidness were 11.6, 11.5 and 11.3, respectively. All cultivars were divided into three groups according to these data (Table 1).

**Table 1.** Evaluation of filling stem by pith of 24 spring wheat cultivars

Group	Genotype	Stem solidness evaluation (points)
Solid	CLTR 7027	23.8
	Carola	23.6
	Tybalt	23.3
	Fortuna	21.5
	Sawtana	21.34
	AC Abbey	21.27
	Tioga	20.7
	Leda Collection A47	20.1
Medium	Glenman	19.6
	Rescue	19.2
	Chinook	18.8
	Alentejano	18.2
	431	17.7
	401	17.5
	Marquis	16.4
	H N ROD 5 13 750	15.3
Hollow	Arabella	13.8
	404	13.6
	Solid Straw Tuscan Varia	11.6
	Americano 44D	11.5
	Ruzynska II	11.3
	Ostka Smolicka	11.1
	Parabola	11.0
	Bombona	5.8

### **The effect of the genotype, pre-treatment of different spikes, hormones used in the induction medium and degree of stem solidness on callus formation and green plant regeneration**

**Figure 1.**
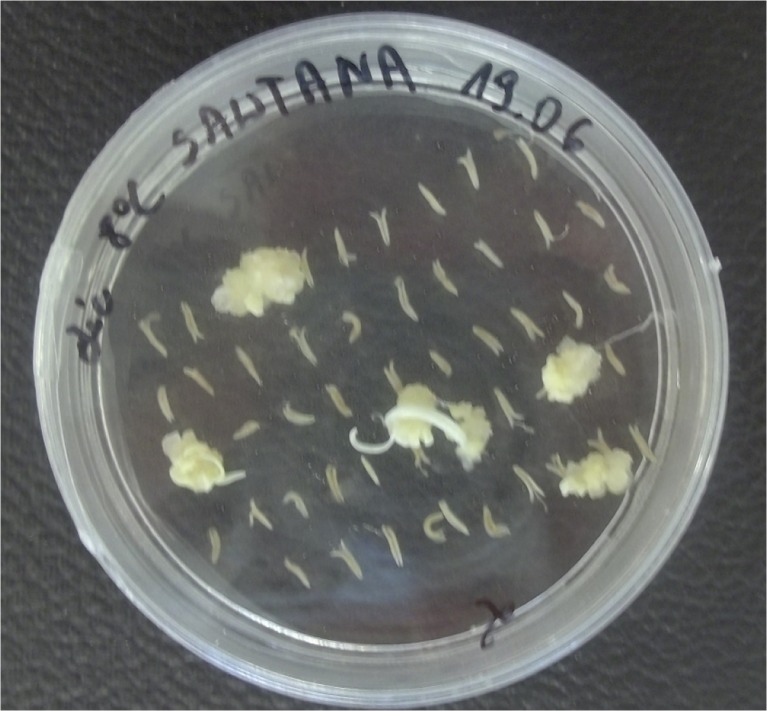
Callus formation from anthers of cv. Sawtana on the induction medium containing 2,4-D and dicamba.

**Figure 2.**
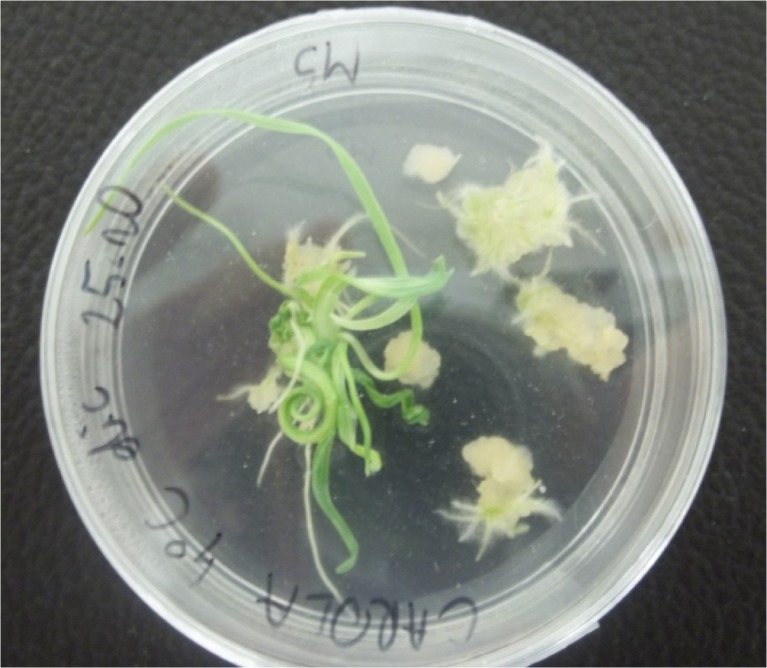
Green plant of cv. Carola regeneration on MS medium.

Calluses were formed after 8 to 10 wk of culturing (Fig. 1). Only the anthers of two cultivars, CLTR 7027 and Bombona, did not form any calluses. The general results of *in vitro* anther culture are shown in Table 2. A total of 43,200 anthers were plated and 1363 calluses were formed. The average efficiency of callus formation was 3.15%. The highest efficiency of callus induction (27.78%) occurred for the cultivar Rescue on medium with 2,4-D and dicamba. A total of 456 plants were regenerated from 1363 calluses (Fig. 2). About 16% of them had chlorophyll defects. Green plants were obtained at an average efficiency of 0.88%, which represented a total of 382 plants. The highest efficiency of green plant regeneration (24%) was observed in the AC Abbey cultivar (solid-stemmed) on medium with 2,4-D and dicamba after cold pre-treatment at 4°C. Green plants were not regenerated from explants of four cultivars: CLTR 7027, Alentejano, Marquis and Bombona. From the calluses of cultivars 401, Solid Straw Tuscan Varia and Parabola, only albino plants were obtained.

**Table 2.** The influence of genotype, temperature pre-treatment and hormones (in the induction medium) on androgenic response of spring wheat

Group	Genotype	2,4-D + dicamba						2,4-D + kinetin					
		CF (no. of calluses/100 anthers)		GPR (no. of green plants/100 anthers)		APR (no. of albino plants/100 anthers)		CF (no. of calluses/100 anthers)		GPR (no. of green plants/100 anthers)		APR (no. of albino plants/100 anthers)	
		4°C	8°C	4°C	8°C	4°C	8°C	4°C	8°C	4°C	8°C	4°C	8°C
Solid	CLTR 7027	0.00	0.00	0.00	0.00	0.00	0.00	0.00	0.00	0.00	0.00	0.00	0.00
	Carola	3.78	1.78	0.22	0.44	0.22	0.00	1.33	1.33	1.33	0.22	0.67	0.00
	Tybalt	2.89	5.11	0.00	4.22	0.00	0.44	1.78	1.11	0.00	0.00	0.22	0.00
	Fortuna	4.67	9.56	1.33	7.33	0.22	1.33	0.00	2.89	0.00	0.89	0.67	0.00
	Sawtana	4.89	5.56	0.67	2.44	0.00	0.00	3.78	3.11	0.44	0.22	0.00	0.44
	Ac Abbey	1.78	4.22	24.00	3.78	0.67	0.22	2.89	5.78	1.33	1.11	0.00	0.00
	Tioga	10.89	14.67	0.67	2.22	0.22	0.22	5.33	8.89	1.11	2.22	0.00	0.00
	Leda Collection A47	2.00	4.44	0.00	1.33	1.11	1.33	0.89	0.89	0.44	0.00	0.44	0.00
Medium	Glenman	3.33	6.67	0.67	0.00	0.00	0.44	3.78	3.78	0.00	1.33	0.00	0.00
	Rescue	0.22	27.78	0.00	0.44	0.22	0.00	1.11	4.44	0.89	2.00	0.00	0.00
	Chinook	4.44	11.56	1.56	0.22	0.22	0.00	4.89	8.22	1.11	1.33	0.22	0.44
	Alentejano	1.56	1.78	0.00	0.00	0.00	0.00	0.00	0.00	0.00	0.00	0.00	0.00
	431	1.11	1.78	0.00	0.00	0.00	0.00	2.00	1.11	0.44	0.00	0.22	0.44
	401	0.00	0.00	0.00	0.00	0.00	0.00	0.89	2.67	0.00	0.00	0.00	0.44
	Marquis	0.44	0.67	0.00	0.00	0.00	0.00	3.11	1.11	0.00	0.00	0.00	0.00
	HN ROD 5 13750	0.67	0.67	0.00	0.44	0.22	0.00	6.00	0.22	0.00	0.00	0.00	0.00
Hollow	Arabella	6.67	5.33	0.67	0.00	1.33	0.67	1.11	2.44	0.00	1.56	0.22	0.44
	404	1.33	2.67	0.00	2.67	0.00	0.89	2.89	1.33	0.00	0.00	0.00	0.00
	Solid Straw Tuscan Varia	0.00	2.00	0.00	0.00	0.00	0.22	1.11	0.89	0.00	0.00	0.00	0.00
	Americano 44	0.44	7.33	0.00	3.33	0.00	0.00	1.11	0.00	0.00	0.00	0.00	0.00
	Ruzynska II	2.00	2.89	0.89	2.44	0.00	0.00	5.33	1.56	3.78	0.44	0.00	0.00
	Ostka Smolicka	2.00	3.11	0.00	0.00	0.44	0.22	0.44	2.00	0.44	0.00	0.00	0.44
	Parabola	2.00	2.22	0.00	0.00	0.00	0.00	2.22	0.89	0.00	0.00	0.22	0.00
	Bombona	0.00	0.00	0.00	0.00	0.00	0.00	0.00	0.00	0.00	0.00	0.00	0.00

The PCA explained 83.6% of the total variability of cultivars in variables under analysis (callus formation, green and albino plant regeneration). As results from the vectors of loadings of variables on the first two principal components, there was a strong positive correlation between the callus formation capacity and green plant regeneration. The variables were not correlated with the occurrence of albino plants.

**Figure 3.**
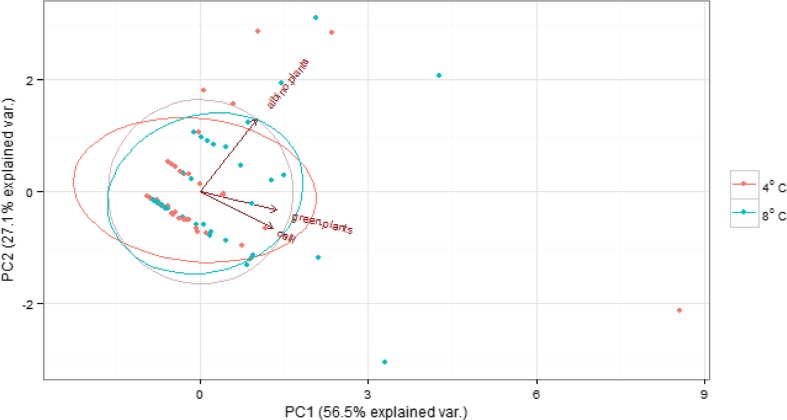
Principal component analysis of the cultivars. Cases are grouped by the applied temperature. *Ellipsoids* indicate 67% confidence.

There were no differences between the cases under observation and response to the temperature applied (Fig. 3). Analogous dependences were also observed in concentration ellipsoids made for temperature-dependent experimental combinations of factors. For this reason this factor was omitted in further analysis.

**Figure 4.**
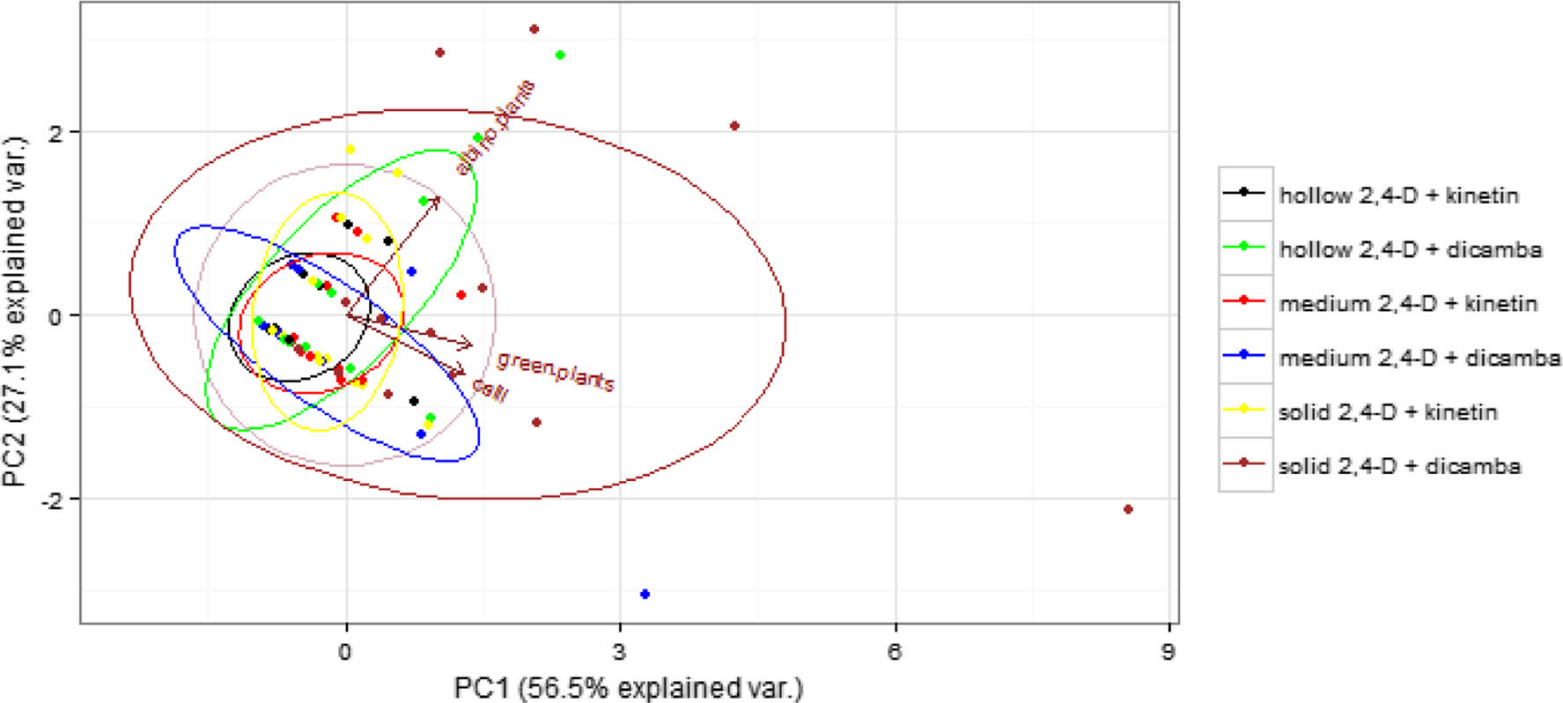
Principal component analysis of the cultivars. Points are grouped by the degree of stem solidness and the medium applied. *Ellipsoids* indicate 67% confidence.

As far as the division according to the degree of stem solidness and the medium applied is concerned, the most calluses and green plants were obtained from the solid stem on the medium containing 2,4-D and dicamba (Fig. 4). In this combination, calluses were formed most efficiently by three cultivars, Rescue, Tioga and Chinook, with callus tissue formation efficiencies of 27.78, 14.67 and 11.56%, respectively (Table 2). The highest efficiency of green plant regeneration (24 per 100 anthers) was obtained from calluses of the cultivar Ac Abbey on C17 medium with 2,4-D and dicamba. Also, there was high efficiency of green plant regeneration from calluses of the cultivar Fortuna (7.33%). Hollow and medium cultivars on the medium with 2.4-D and kinetin resulted in similar values of variables, wherein the abundance of green plants in the medium group was higher than in the hollow one. Anthers of the cultivar Alentejano produced calluses only on the induction medium with 2,4-D and dicamba, while anthers of genotype 401 produced calluses only on the induction medium C17 with 2,4-D and kinetin. Calluses from four cultivars (Tybalt, HNROD 513750, 404 and Americano 44D) regenerated green plants only on the induction medium with 2,4-D and dicamba. On the contrary, green plants of cultivars 431 and Ostka Smolicka were obtained only from calluses produced on C17 medium with 2,4-D and kinetin. The majority of albino plants occurred for the hollow cultivars on the medium with 2,4-D and dicamba and for solid cultivars on the medium containing 2,4-D and kinetin (Fig. 4).

## Discussion

Solid-stemmed wheat cultivars were developed to minimize losses from wheat stem sawflies and lodging. Cultivar S-615 is the source of solidness of the stem, the presence of which can be found in most of the cultivars from North America. Polish cultivars are characterized by hollow stems, so solidness would be an improvement. To accelerate this process, androgenesis in anther cultures can be used. Unfortunately, the cultivars which are important in agriculture are often recalcitrant in these techniques. Zheng (2003) suggested that the ability to induce androgenesis is an inherited and variable trait. Dağüstü (2008) and Kondić-Špika *et al.* (2008) suggested that androgenesis should only be applied for breeding combinations in which at least one of the parent lines has high androgenic capacity. Recalcitrant but valuable cultivars can be crossed with varieties which respond to androgenesis well (Kim *et al.* 2003). Therefore, the ability of genetic improvement by androgenesis may be more useful than the manipulation of external factors (Dağüstü 2008).

The genotype of the donor plant is a major factor influencing the ability to induce androgenesis (Rybczyński *et al.* 1991; Ayed *et al.* 2010; Tadesse *et al.* 2012). The present study confirmed this observation. Among 24 cultivars used in the experiment there were considerable differences both in the callus formation efficiency (ranging from 0 to 27.78%) and the green and albino plant regeneration (ranging from 0 to 24% and from 0 to 1.33%, respectively). The best efficiency of green plant regeneration was obtained from AC Abbey cultivar, which served as control across our previous experiments. Similar efficiencies were noted in a previous study on spring wheat cultivars resistant to *Fusarium*, in which the average regeneration efficiency was genotype-dependent and ranged from 0 to 17.33% (Weigt *et al.* 2012).

Despite strong genotypic differences, it is possible to obtain haploid plants through androgenesis even from recalcitrant cultivars by appropriate crossing or by adjustment of pre-treatment conditions and composition of the culture medium (Trottier *et al.* 1993; Datta 2005; Redha and Talaat 2008). The induction of changes in a microspore development from the gametophytic to sporophytic pathway often requires a stress stimulus. Delaying the mitosis in pollen by cooling or other factors is beneficial to the induction of androgenesis (Śnieżko 1991; Shariatpanahi *et al.* 2006). Thermal pre-treatment is the most common factor stimulating androgenesis (Murovec and Bohanec 2012). Pre-treatment of spikes at low temperatures (3 to 10°C for 5 to 14 d) is the most common method of wheat pollen induction (Śnieżko 1991; Wang *et al.* 2000). The present study examined the influence of two different cold pre-treatments (4 and 8°C) on the induction of androgenesis and green plant regeneration. As the statistical analysis revealed, there were no differences in response between these two temperatures. Similar thermal shock conditions were proposed by El-Hennawy *et al.* (2011). They applied a cold pre-treatment at 4°C for 6 to 8 d and observed green plant regeneration efficiency of 0 to 10%. Datta (2005) recommends wheat spike treatment at a higher temperature (8°C) for 10 d.

In most plant species growth regulators are a very important factor stimulating cell division in pollen. An exogenous auxin is necessary to induce androgenesis. The present study showed an almost doubling of callus formation and tripling of green plant formation on a medium supplemented with 2,4-D and dicamba, as compared with a medium supplemented with 2,4-D and kinetin. Satyavathi *et al.* (2004) also observed the beneficial effect of dicamba. Hassawi *et al.* (1999) observed the highest callus regeneration on a medium supplemented with 2,4-D and kinetin in comparison with a medium containing 2,4-D and dicamba. It appears that cultivars of various origins can react differently to the composition of hormones in the inducing medium. This assumption is confirmed not only by the referenced publications but also by previous research from this research group (Weigt *et al.* 2012).

The present study strongly indicates that a relationship between androgenic capacity and stem solidness exists. Solid-stemmed cultivars produced more calluses and regenerated more green plants than the cultivars with a medium or hollow stem. This is the first report about the relationship between the content of pith in the stem and plant androgenic capacity. The interdependence between stem solidness and the capacity to induce androgenesis will facilitate the production of new wheat varieties with pith in stems by using *in vitro* cultures.
